# Plant Organ Shapes Are Regulated by Protein Interactions and Associations With Microtubules

**DOI:** 10.3389/fpls.2018.01766

**Published:** 2018-12-04

**Authors:** Mark D. Lazzaro, Shan Wu, Ashley Snouffer, Yanping Wang, Esther van der Knaap

**Affiliations:** ^1^Department of Biology, College of Charleston, Charleston, SC, United States; ^2^Center for Applied Genetic Technologies, University of Georgia, Athens, GA, United States; ^3^Boyce Thompson Institute, Cornell University, Ithaca, NY, United States; ^4^National Engineering Research Center for Vegetables, Beijing Academy of Agriculture and Forestry Sciences, Beijing, China; ^5^Institute for Plant Breeding, Genetics and Genomics, University of Georgia, Athens, GA, United States; ^6^Department of Horticulture, University of Georgia, Athens, GA, United States

**Keywords:** OFP, TRM, SUN, IQD, microtubules, organ shape

## Abstract

Plant organ shape is determined by the spatial-temporal expression of genes that control the direction and rate of cell division and expansion, as well as the mechanical constraints provided by the rigid cell walls and surrounding cells. Despite the importance of organ morphology during the plant life cycle, the interplay of patterning genes with these mechanical constraints and the cytoskeleton is poorly understood. Shapes of harvestable plant organs such as fruits, leaves, seeds and tubers vary dramatically among, and within crop plants. Years of selection have led to the accumulation of mutations in genes regulating organ shapes, allowing us to identify new genetic and molecular components controlling morphology as well as the interactions among the proteins. Using tomato as a model, we discuss the interaction of Ovate Family Proteins (OFPs) with a subset of TONNEAU1-recruiting motif family of proteins (TRMs) as a part of the protein network that appears to be required for interactions with the microtubules leading to coordinated multicellular growth in plants. In addition, *SUN* and other members of the IQD family also exert their effects on organ shape by interacting with microtubules. In this review, we aim to illuminate the probable mechanistic aspects of organ growth mediated by OFP-TRM and SUN/IQD via their interactions with the cytoskeleton.

## Introduction

Plant organs display remarkable phenotypic diversity among and within species. Especially for cultivated crops, selection for the harvestable organs has led to greatly increased size and variable shapes of the produce. This diversity is critical for the successful marketing of a wide array of foods such as fruits, vegetables, seeds, leaves, and tubers. Recent studies have revealed many genes that control the growth form of agriculturally important organs (Zuo and Li, 2014; van der Knaap and Ostergaard, 2017). This includes a newly discovered genetic pathway, which through protein interactions and associations with microtubules is proposed to lead to changes in cell division patterns that accompany the different growth forms (Wu et al., 2018). Mechanistically, how the growth forms are controlled by these genes is largely unknown.

The classification of varieties of the same crop based on morphological descriptors is critical. Organizations such as the Union for the Protection of New Varieties of Plants^1^ and the International Plant Genetic Resources Institute (IPGRI) ^2^ developed descriptors of the shape of many vegetables and fruits such that varieties can be distinguished from one another. These descriptors have become the framework for the identification of genes underlying the morphological variation in crops like tomato (Brewer et al., 2006; Rodriguez et al., 2011a). Consumers recognize the shape and size of vegetables and fruits for the different culinary purposes and/or cultural significances (Pickersgill, 2007; Daunay et al., 2008; De Haan, 2009; Monforte et al., 2014). Similarly important for grains, the slender rice grain shape is associated with improved transparent appearance and reduced undesirable grain quality and is, therefore, highly sought-after in certain cuisines (Calingacion et al., 2014; Harberd, 2015).

## Processes That Control Organ Morphology

Lateral plant organs such as leaves and fruits typically initiate in the flanks of apical meristems. Together with the hormone auxin, *AGAMOUS* (for ovaries/fruits) and *CUP SHAPED COTYLEDON/NO APICAL MERISTEM* initiate organ primordia by setting up organ identity and boundaries (Maugarny-Cales and Laufs, 2018). To change from a meristematic cell fate to an organ fate, the down regulation of KNOXI transcription factors by ASYMMETRIC LEAVES1 and LATERAL ORGAN BOUNDARIES DOMAIN proteins is required (Maugarny-Cales and Laufs, 2018). Many hormones play important roles in the growth of organs, in particular gibberellins and brassinosteroids.

**FIGURE 1:**
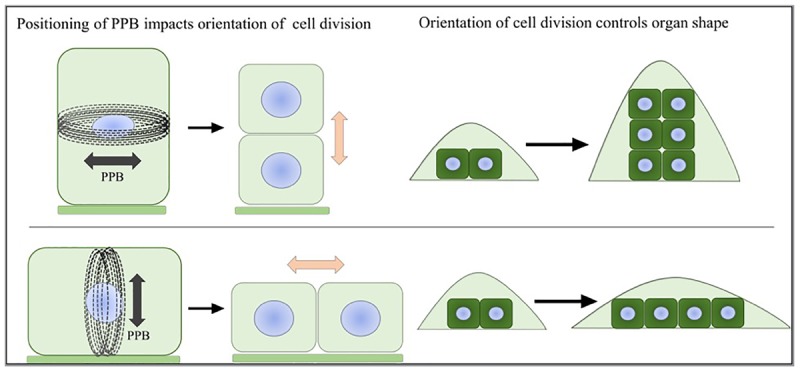
Preprophase band and organ shape. The positioning of the PPB marks the future site of cell division. The direction of cell division will greatly influence the shape of the emerging organ.

The patterns of further outgrowth occur along different axes: the proximal-distal, the medial-lateral and the abaxial-adaxial axis (Van der Knaap et al., 2014). Simply stated, isotropic growth along all three axes tends to lead to larger and round shapes whereas anisotropic growth leads to alternate shapes. For multidimensional organs such as the fruit, the different tissue types grow in an anisotropic way and together form an overall spherical or elongated shape (van der Knaap and Ostergaard, 2017). At the cellular level, the growth patterns are manifested by a combination of cell proliferation (growth and division) and cell enlargement (growth without cell division) driven by turgor pressure. The directions of cell enlargement are guided and restricted by cellulose microfibrils, which are glucose polymers bundled together by hydrogen bonds and Van der Waals forces. These polymers are deposited into the cell wall by CSCs guided by cMTs, In cells undergoing anisotropic expansion, cellulose microfibrils are deposited perpendicular to the axis of expansion and are coaligned with cMTs (Szymanski and Cosgrove, 2009; McFarlane et al., 2014). During cell proliferation, the plane of cell division is determined by the positioning of the preprophase band (PPB) (Rasmussen et al., 2011). The duration and rates of cell proliferation also affect the pattern of growth. Since plant cells are bound to surrounding cells by cell walls, once division has taken place, including formation of the phragmoplast, plant cells are positioned in the same relative location as when they were formed. Therefore, the orientation of cell division has a dramatic effect on the final shape of plant organs (Figure 1; Meyerowitz, 1997; Jenik and Irish, 2000; Van Damme et al., 2007; Schaefer et al., 2017). Mechanical forces provide direct signals leading to coordinated growth toward the final organ shape and size (von Wangenheim et al., 2016). During lateral organ initiation, a highly organized supracellular alignment of microtubule arrays forms along the maximal stress in the region between the meristematic dome and lateral primordia (Hamant et al., 2008). The microtubules guide the directional deposition of cellulose microfibrils, which reinforces the cell wall strength along the appropriate axes to separate the new organs and the undifferentiated cells. During growth, microtubule array dynamics are regulated to respond to the mechanical forces (Uyttewaal et al., 2010). The reorientation of microtubule arrays along the maximal tensile stress can control the directions of cell division and cell expansion leading to heterogeneous growth.

Understanding the molecular basis of shape of harvestable organs comes mostly from studies conducted in tomato and rice. The increase in rice grain size is often accompanied with altered shape, and found to be under the control of proteins involved in diverse pathways such as G-protein signaling, the ubiquitin–proteasome pathway, phytohormone signaling including brassinolides, auxin and cytokinin, as well as transcriptional regulation (Zuo and Li, 2014; Zheng et al., 2015). In the case of tomato, the identified proteins appear functionally less diverse as they seem to interact with the cytoskeleton. Specifically, a mutation in *OVATE*, the founding member of the OFP class, and another member named *SlOFP20* result in a distinct pear shaped tomato fruit (Wu et al., 2018). OVATE and SlOFP20 interact with several members of the Tonneau1 Recruitment Motif (TRM) proteins, which are often found associated with microtubules (Hamant et al., 2008; Wu et al., 2018). SUN, a member of the IQ Domain (IQD) family, also impacts tomato fruit shape (Xiao et al., 2008). Members of the IQD family have been found to interact with calmodulin (CaM) as well as microtubule binding proteins Kinesin Light Chain-Related protein-1 (KLCR1) and SPR2 to regulate microtubule structure based on external auxin and calcium inputs (Burstenbinder et al., 2013, 2017a,b; Wendrich et al., 2018; Yang et al., 2018).

## The Role of OFPs, SUNs and TRMs on Organ Shape

### OFP and SUN

**FIGURE 2:**
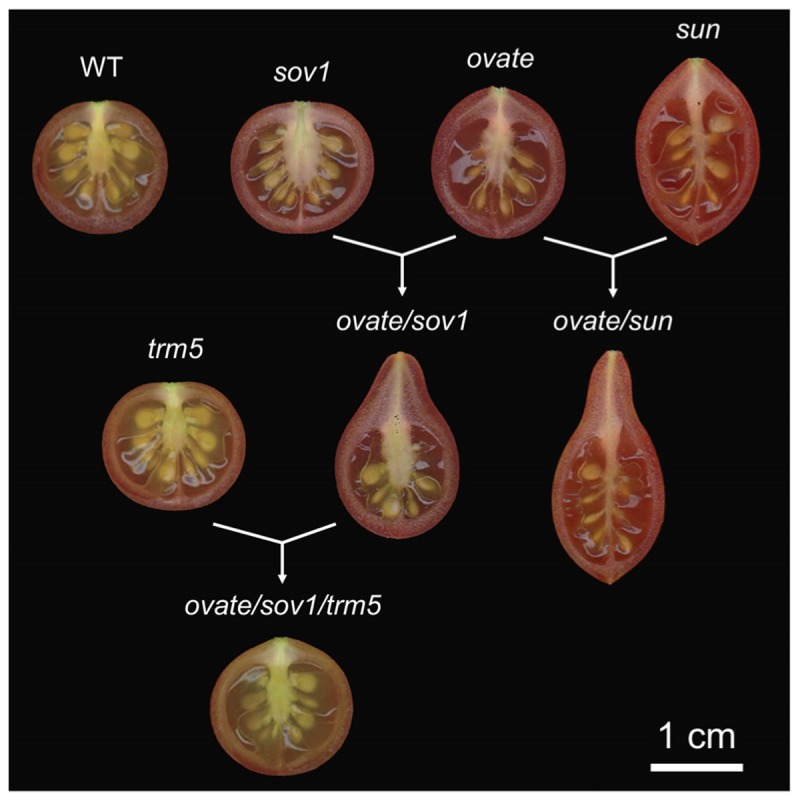
Effect of the fruit shape genes in the wild type tomato background. The loci were introgressed (*sun, ovate, sov1*) or edited (*trm5*) in the *Solanum pimpinellifolium* accession LA1589 background to create near isogenic lines (NILs). WT, wild type; *sov1, suppressor of ovate* corresponding to *SlOFP20*. The single natural NILs are shown on the top of the figure, whereas the double and triple NILs are shown below the single NILs.

*OVATE* and *SUN* are two important genes controlling tomato fruit shape. The shape of many oval shaped varieties, including grape tomatoes, is controlled by *OVATE*. *SUN* can be found in very elongated, tapered or oxheart shaped heirloom as well as commercially grown plum tomatoes (Ku et al., 1999; Liu et al., 2003; Rodriguez et al., 2011b; Van der Knaap et al., 2014). *OVATE* is the founding member of the OFP class. Recently a new fruit shape gene was identified as a suppressor of *ovate* (*sov1*). This fruit shape gene is another member of the same family, *SlOFP20* (Huang et al., 2009; Rodriguez et al., 2013; Wu et al., 2018). Whereas *ovate* is a null, the *Slofp20* allele shows reduced expression and the effects of both mutations on ovary shape are already apparent at anthesis (Van der Knaap and Tanksley, 2001; Van der Knaap et al., 2014; Wu et al., 2018). This finding implies that the patterning mediated by these *OFP*s occurs early in the ontogeny of the ovary, perhaps immediately after organ initiation. *SUN* also affects ovary shape before anthesis and continues to promote fruit elongation immediately after fertilization (Van der Knaap and Tanksley, 2001; Xiao et al., 2009; Wu et al., 2011). *sun* is due to a transposon-mediated duplication event leading to high expression of the transposed gene during reproductive development (Xiao et al., 2008). Over-expression of *SUN* in both wild and cultivated tomatoes leads to evenly elongated fruit shape (Xiao et al., 2008). Interestingly, *sun* synergistically interacts with *ovate* and together the two promote growth at the proximal end to form a pear-shaped and pointed tomato fruit (Wu et al., 2015; Figure 2). *ovate* and *sov1* also synergistically interact to form a pear-shaped tomato but with a round bottom shape (Wu et al., 2018; Figure 2). This suggests that obovoid organ shapes may be achieved by alleles from different sets of proteins or that the pathways intersect.

**FIGURE 3:**
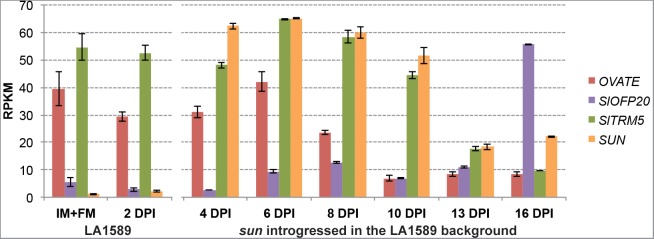
Expression patterns of *OVATE, SlOFP20, SlTRM5* and *SUN* during floral development. Samples were collected from wild-type *S. pimpinellifolium* LA1589 or the *sun* NIL in the LA1589 background. RPKM, reads per kilobase of transcript per million mapped reads; IM, inflorescence meristem; FM, floral meristem; dpi, flower buds collected in number of days post floral initiation. Each value represents 3 to 4 biological replicates, each containing 100–150 meristems or young flower buds. The bars indicate standard errors among the four replicates. The expression data is available under BioProject number PRJNA343236 and SRP089970 as well as on the Sol Genomics Network website (https://www.sgn.cornell.edu/) at the Tomato Functional Genomics Database (http://ted.bti.cornell.edu/cgi-bin/TFGD/digital/home.cgi).

The expression of wild type *OVATE* in tomato is high in the IM/FM, and its expression reduces 8 days after floral initiation (dpi) (Figure 3). In contrast, the expression of wild type *SlOFP20* is relatively low in the IM/FM and increases in 6 dpi buds, with a dramatic increase at 16 dpi (Figure 3). For *SUN*, wild type gene expression is very low (first two time points, LA1589) whereas in the NIL with the retrotransposon-mediated duplication (*sun* introgressed in the LA1589 background), *SUN* is highly expressed during floral development (Figure 3). The initiation of the gynoecium primordia occurs at 6 dpi (Xiao et al., 2009), which is when *OVATE, SlOFP20* and *SUN* are well expressed. At 8 and 13 dpi, the expression of *OVATE* and *SUN* respectively, is much reduced from expression levels at the earlier developmental stages coinciding with when these genes may function in development.

*OVATE, SlOFP20* and *SUN* affect neither floral organ identity, nor the organization or number of floral organs (Wu, 2015). Instead, *SUN, OVATE* and *SlOFP20* regulate organ elongation by altering the directions of cell division along the proximal-distal axis (Wu et al., 2011, 2018). Whereas *SUN* affects cell division along the entire proximal-distal axis, *OVATE* and *SlOFP20* appear to have a specific role in anisotropic growth primarily at the proximal end of the ovary. In NILs that carry the *ovate* and *sov1* mutant alleles, there is an increased number of cells in the proximal-distal direction and a reduced number of cells in the medial-lateral direction compared to wild type. Cell size is also enlarged but cell shape appears to change little in the mutant background (Wu et al., 2018). Thus, the effect of cell size and shape in fruit elongation is not clear and therefore, cell division patterns are thought to drive the shape of the *ovate/sov1* fruits.

Certain *OFP*s and *SUNs* are likely to be involved in conserved mechanisms of morphology regulation across plant species. Genetic evidence indicates that the same subclade of OFPs, represented by Arabidopsis *OFP1* and tomato *OFP20*, controls tomato fruit shape as well as aerial organ shapes in Arabidopsis (Wang et al., 2011), tuber shape in potato, and fruit shape in melon (Wu et al., 2018). Specifically, the potato (*Solanum tuberosum* L.) tuber shape QTL *Ro* has been fine-mapped in an outcrossing diploid F_1_ population to a region on chromosome 10 that contains the potato ortholog of *SlOFP20.* There is also strong association between tuber shape and *StOFP20* in a separate inbred diploid F_2_ population. Very elongated tubers lack the *StOFP20* gene, consistent with its role in the regulation of organ shape as found in tomato. In melon (*Cucumis melo*), fine mapping within the fruit shape QTL *fsqs8.1* has identified *CmOFP13* in a cross of Piel de Sapo and PI124112 (Wu et al., 2018). For *SUN* and other members of the *IQD* family, natural mutations affecting organ shape have been found in rice and species in the Cucurbitaceae family. Specifically, the rice gene *GSE5* at the *GW5/qSW5* locus encodes a SUN member closely related to Arabidopsis IQD25-27 (Duan et al., 2017). The change in grain shape is due to increased cell proliferation in spikelet hulls. Interestingly, in cucumber and watermelon, a SUN member that is also most similar to Arabidopsis IQD25-27 likely controls fruit shape in these two species (Pan et al., 2017; Dou et al., 2018). Another rice SUN-like gene, *OsIQD14* has been shown to affect rice grain shape and this member is most closely related to another subclade of the *SUN/IQD* family (Yang et al., 2018). Arabidopsis, overexpression of several IQD members leads to altered organ shapes. The overexpression of microtubular localized AtIQD16 and AtIQD11 resulted in elongated aerial organs with left-handed helical growth abnormalities similar to the phenotype of the tomato *SUN* overexpressors (Wu et al., 2011; Burstenbinder et al., 2017b). Overexpression of *AtIQD14* results in organ twisting but not cell elongation as observed in *AtIQD11* and *AtIQD16* (Burstenbinder et al., 2017b), a phenotype that resembles that of *tortifolia*/*spiral* mutants (Furutani et al., 2000; Buschmann et al., 2004; Shoji et al., 2004). Furthermore, overexpression of plasma membrane localized *IQD25* resulted in rounder leaves and larger cells, the opposite phenotype from that observed in overexpression of microtubule localized IQDs suggesting that IQD proteins can have diverse functions in regulating the cytoskeleton and cell elongation (Burstenbinder et al., 2017b). Thus, in addition to *SUN* in tomato, several members of this family have been associated with changing plant organ shape.

### TRMs

A knockout mutation in tomato’s TONNEAU1 Recruiting Motif 5 (*SlTRM5*) results in a slightly flatter fruit yet its effect is most strongly noticeable in the *ovate/sov1* mutant background (Figure 2). The expression of wild type *SlTRM5* is high in IM/FM throughout floral development until 13 dpi (Figure 3), following similar expression dynamics as *OVATE* and *SUN*. *SlTRM5* is a member of the Arabidopsis *TRM1-5* subclade in which tomato carries only two *TRM* paralogs. At the cellular level, SlTRM5 controls the number of cells in the proximal-distal and medial-lateral direction such that the mutant allele *Sltrm5* rescues the tomato fruit shape phenotype of *ovate/sov1*. *TRM5* orthologs and close paralogs also appear to regulate organ shape in other crops. For example, the cucumber ortholog of *TRM5* underlies the *fs2.1* QTL controlling fruit shape (Wu et al., 2018). In rice, a major QTL for grain length encodes a TRM member in the TRM1-5 subclade. The discovery was made in three independent studies as *GRAIN LENGTH ON CHROMOSOME 7* (*GL7*)/*GRAIN WIDTH 7* (*GW7*)/*SLENDER GRAIN ON CHROMOSOME 7* (*SLG7*) loci (Wang Y. et al., 2015; Wang S. et al., 2015; Zhou et al., 2015). Copy number variants at the *GL7* locus contribute to grain size diversity (Wang Y. et al., 2015) and the increased expression of *GW7/SLG7* increases grain length (Wang S. et al., 2015; Zhou et al., 2015). However, these studies show contrasting effects on the cellular mechanisms of grain shape changes. Higher expression of *GW7* increased cell division in the proximal-distal direction and decreased cell division in the medial-lateral direction (Wang S. et al., 2015), which is similar to the effect of *SlTRM5* on tomato fruit shape. On the other hand, increased expression of *SLG7* increased cell length and decreased cell width with no changes in cell division (Zhou et al., 2015). In Arabidopsis, certain members of the *TRM1-5* subclade control the elongation of various aerial organs. Overexpression of *AtTRM1* (*LONGIFOLIA2*) or *AtTRM2* (*LONGIFOLIA1*) leads to extremely long cotyledons, leaves, floral organs and siliques (Lee et al., 2006). On the other hand, loss-of-function mutations in *AtTRM1* or *AtTRM2* cause shortened siliques and cotyledons (Lee et al., 2006; Drevensek et al., 2012), which intriguingly mimic the phenotypes of *AtOFP* overexpressors (Wang et al., 2007). The more elongated leaf blades seen in the *AtTRM1* and *AtTRM2* overexpressors are due to increased cell expansion along the proximal-distal axis rather than an altered cell proliferation pattern (Lee et al., 2006).

## Mechanistic Insights Into the Regulation of Organ Shape

### Interaction Between OFPs and TRMs

As mentioned in the previous section and based on several studies, TRMs play a critical role in regulating organ shape. In tomato, TRMs were first discovered in a Yeast 2-Hybrid (Y2H) experiment using OVATE as bait. The goal of the experiment was to identify molecular interactants of OVATE to learn about cell division patterning mediated by OFP family members. A total of 11 out of 26 members of the TRM superfamily were identified in the screen. What set these OVATE-interactants apart from the other members of the TRM family was the conserved M8 motif (Van der Knaap et al., 2014; Wu et al., 2018). These findings suggest that the genetic interaction of TRM5 is through protein-protein interactions with OVATE via the TRM M8 motif. To validate the findings from Y2H, the interaction motifs were mapped in OVATE, SlOFP20, and several Ovate-interacting TRMs (Wu et al., 2018). OVATE and SlOFP20 both interact through highly conserved negatively charged amino acids in the OFP domain with TRMs via the highly conserved basic residue (K or R) in the TRM M8 motif. It is reasonable to conclude that the electrostatic interactions in the OFP domain and the M8 motif enable the interactions between these proteins (Wu et al., 2018).

**FIGURE 4:**
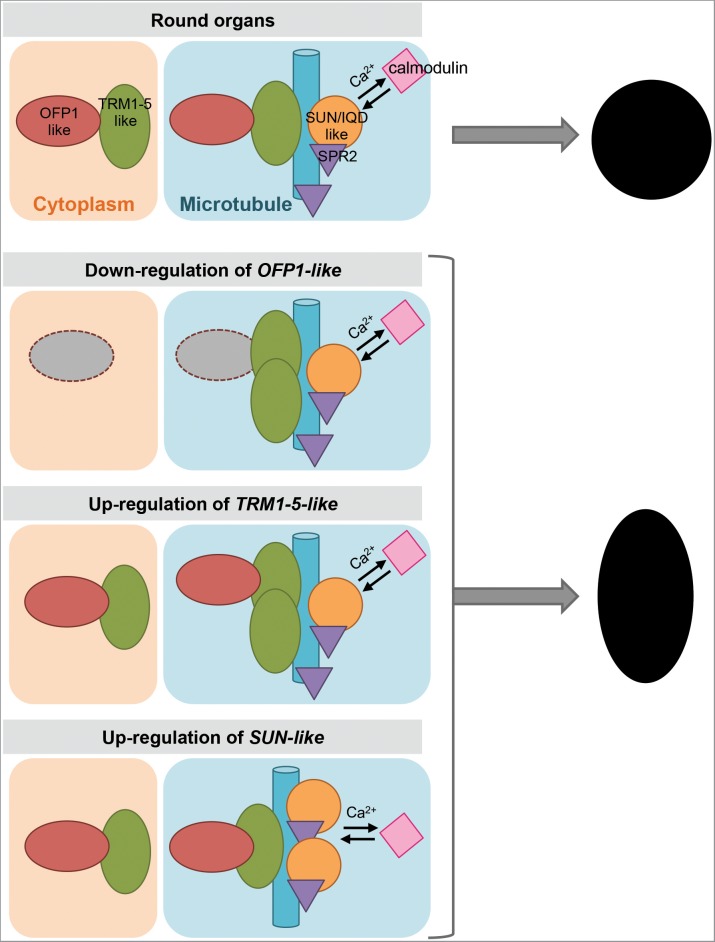
A model of the regulation of plant organ shapes. Expression levels of OFP1-like, TRM1-5-like and SUN/IQD-like lead to more or less association with microtubules to determine organ shape. Red oval shape, OFP1-like; green oval shape, TRM1-5-like; blue bar, microtubule; orange circle, SUN/IQD-like; purple triangle, SPR2; pink square, calmodulin.

The Y2H protein interactions have also been validated in a plant system. OVATE, SlOFP20 and several Ovate-interacting TRMs were expressed as fusion proteins in *Nicotiana benthamiana* leaf epidermal cells (Wu et al., 2018). When expressed alone, OVATE localizes in the cytoplasm and SlOFP20 is in the nucleus and cytoplasm. When SlTRM3/4 or SlTRM5 (members of the AtTRM1-5 subclade) are expressed alone, they localize to microtubules. Co-expression of OVATE and SlTRM5 dissociates SlTRM5 from microtubules and both proteins are found in the cytoplasm. On the other hand, co-expression of SlOFP20 and SlTRM5 causes the localization of SlOFP20 to microtubules coincident with SlTRM5. Co-expressions of OVATE or SlOFP20 with SlTRM3/4 both lead to a nearly complete dissociation of SlTRM3/4 from microtubules to the cytoplasm (Wu et al., 2018). These re-localizations are much reduced when mutants of OVATE, SlOFP20, SlTRM5, and SlTRM3/4 lacking the interacting charged amino acid residues are co-expressed in *N. benthamiana* cells. These findings imply that relocalization occurs through physical protein interactions. Bifluorescence complementation assays further demonstrate that the charged amino acid residues of OVATE and SlTRM5, or OVATE and SlTRM3/4, are responsible for their interactions as well as relocalizations (Wu et al., 2018). The relocalization of OFPs and TRMs to different subcellular compartments upon interaction suggests that a dynamic balance between cytoplasmic- and microtubular-localized OFP-TRM protein complexes regulates cell division and organ growth. A mechanistic model describing the function of OFPs and TRMs to control organ shape is shown in Figure 4.

The subcellular relocalization when co-expressing OVATE or SlOFP20 on the one hand and SlTRM5 or SlTRM3/4 on the other hand suggests that certain OFPs play a critical role in localizing protein complexes. The discovery of the OFP-TRM module may provide an explanation for how other OFPs would serve as regulators in various distinct developmental processes by their effect on subcellular localization. AtOFP5 negatively affects the function of BLH1-KNAT3 complex in early embryo sac development (Pagnussat et al., 2007). The authors show that this is due to abnormal migration and positioning of embryo sac nuclei during megagametogenesis. They propose that this could be due to a change in the behavior of microtubules (Pagnussat et al., 2007) that serve as tracks for nuclear movement in plant cells (Vogelmann et al., 1981; Meindl, 1983; Mineyuki and Furuya, 1985). However, no evidence was available at that time to link the function of AtOFP5 with microtubule dynamics. Another example is offered by AtOFP4 that interacts with KNAT7 to regulate secondary cell wall formation (Li et al., 2011). cMTs participate in secondary cell wall development by directing the deposition of cell wall matrix components (Oda et al., 2005). The defects in secondary cell wall formation could be caused by an abnormal microtubule behavior or due to mislocalization of the CSCs due to the loss-of-function of AtOFP4. Again, this suggests that subcellular localization of CSCs may be disrupted by an OFP. Interestingly, SUN-like protein, AtIQD13 is also associated with positioning the CSCs by influencing the organization of the cMT arrays that guide them (Sugiyama et al., 2017). Other research has shown that the BEL1-like homeodomain 1 (BLH1) protein is shuttled from the nucleus to the cytoplasm when interacting with AtOFP1 or AtOFP5. It was therefore proposed that AtOFPs affect the activities of TALE transcription factors by altering their subcellular localization (Hackbusch et al., 2005). AtOFP1 was also identified as a protein partner of AtKu70, which plays a role in non-homologous end-joining DNA repair (Wang et al., 2010). Interestingly, the centromeric function of Ku70 depends on the presence of microtubules (Cabrero et al., 2013). Therefore, it is reasonable to propose that AtOFP1 may function in DNA-repair by affecting the anchoring of AtKu70 to microtubules. Thus, the OFP-TRM module could explain these seemingly unrelated pathways where OFP controls the subcellular localization of protein complexes. This idea is in stark contrast with the notion in the literature that AtOFPs are transcriptional repressors (Wang et al., 2007, 2011). However, these conclusions were primarily made based on protoplast expression assays as well as expression correlation when overexpressing *AtOFP1* (Hackbusch et al., 2005; Wang et al., 2007) and thus transcriptional repression was not validated in intact plants.

### TRMs, TTP and the Cytoskeleton

A broader function of the TRMs is their role in assembling the TTP (TON1-TRM-PP2A) complex. Before members of this family were associated with the TTP complex, two members namely LONGIFOLIA (LNG) 1 and LNG2, were identified to control organ shape in Arabidopsis (Lee et al., 2006). The entire TRM family, however, was identified in Arabidopsis in a Y2H study using TON1 as bait (Drevensek et al., 2012). The Arabidopsis TRMs consists of 34 members and all contain the TON1-interacting M2 motif at the C terminus. A typical TRM in Arabidopsis is AtTRM1, a microtubule-associated protein that localizes to cMTs *in vivo* and binds microtubules *in vitro*. AtTRM1 recruits TON1 to microtubule arrays in *N. benthamiana* leaf cells. A subset of TRMs target the TTP complex to microtubules (Drevensek et al., 2012). The TTP complex has been proposed to regulate the organization of microtubule arrays and PPB formation, and thus cell division patterns and cell growth (Camilleri et al., 2002; Azimzadeh et al., 2008; Drevensek et al., 2012; Spinner et al., 2013; Schaefer et al., 2017). Throughout interphase in plant cells, microtubules are found just beneath the plasma membrane in the cell cortex. These cMTs determine cell shape as they form patterns in the absence of focused nucleation centers like centrosomes in animal and fungal cells. The nucleation of new cMTs is geometrically constrained (Fishel and Dixit, 2013). Most initiate from a nucleation site on the side of a parent microtubule. These nucleation sites contain γ-tubulin and associated γ-tubulin complex proteins (Nakamura et al., 2010; Murata and Hasebe, 2011) to form the γ-TuRC. After nucleation, new microtubules elongate at about 40^o^ from the parent microtubule (Chan et al., 2009; Nakamura et al., 2010; Murata and Hasebe, 2011). New microtubules also grow parallel to the parent microtubule and move alongside existing microtubules by polymer treadmilling (Chan et al., 2009; Nakamura et al., 2010). The branched form of nucleation is the dominant pattern, while parallel nucleation occurs about half as frequently. About 1–2% of nucleation events occur *de novo*, where a new microtubule elongates independently of an existing microtubule (Shaw et al., 2003; Chan et al., 2009; Nakamura et al., 2010). As a component of the TTP complex, the *Arabidopsis thaliana* B” subunit of protein phosphatase 2A is encoded by the *TONNEAU2/FASS* (*TON2*) gene (Camilleri et al., 2002), and microtubule branching nucleation is specifically promoted by this regulatory subunit (Kirik et al., 2012). In *ton2-15* mutants, the frequency of microtubule branching nucleation is reduced 4-fold while the frequency of parallel nucleation is increased 2.4-fold. The branching angle of new microtubules is unchanged. In hypocotyl cells, loss of *TON2* function also results in the inability of microtubule arrays to reorient in response to light, suggesting an essential role for *TON2* and microtubule branching nucleation in the reorganization of microtubule arrays (Kirik et al., 2012). It has been postulated that TON2 may influence the orientation of initial polymerization through a direct interaction with or phosphorylation of a component of the γ-TuRC (Fishel and Dixit, 2013). Microtubule assembly at the γ-TuRC is also partly regulated by the microtubule severing protein katanin (Nakamura et al., 2010) and katanin1-mediated microtubule rearrangement is proposed to play a role in regulating rice grain shape controlled by a SUN-like gene, *OsIQD14* (Yang et al., 2018). This finding supports the notion that the regulation of organ shape might be functionally linked by the OFP-TRM and SUN/IQD pathways.

The formation and function of different microtubule arrays are regulated by microtubule nucleation, dynamics and stability. Microtubule assembly is a polarized process starting from one or several MTOCs (Murata and Hasebe, 2011). The centrosome is the major MTOC in animal cells to recruit and modify cell cycle proteins (Wu and Akhmanova, 2017). Even though vascular plant cells lack centrosomes, TTP components have sequence similarity to animal centrosomal proteins. For example, the N-terminus of TON1 has sequence similarity to FOP and OFD1, proteins required for microtubule anchoring and stability within the centrosome, respectively (Yan et al., 2006; Azimzadeh et al., 2008; Singla et al., 2010). In addition, three TRM motifs (i.e., M3-M4-M2) are found in the human centrosomal protein CAP350, which interacts with FOP. The C-terminal M2 motif in CAP350 is responsible for FOP recruitment to the human centrosome and facilitates microtubule anchoring within the centrosome (Yan et al., 2006). Similarly, TON1 and the TTP complex bind to the PPB, which marks the future division plane by promoting spindle bipolarity and limiting spindle rotation to ensure properly patterned cell division. The consequence of the sequence similarity and overlapping motifs between TTP complex proteins and animal centrosomal proteins may be the functional similarities among the complexes in plant and animal cell division (Schaefer et al., 2017). The TTP complex is required for proper PPB assembly and division plane establishment (Spinner et al., 2013). The PPB is an array of microtubules and actin filaments that forms a ring at the cell periphery during G2 and persists throughout prophase. Although the PPB is disassembled as the nuclear envelope breaks down and the mitotic spindle forms, its position precisely correlates with the position of the future division plane. The spatial information of the PPB is preserved by selective recruitment and depletion of proteins that lead to the generation of the cortical division zone and the precise positioning of the cell plate during cytokinesis (Rasmussen et al., 2011; Rasmussen and Bellinger, 2018). TON1A, TON1B and the PP2A subunit FASS/TON2 (in Arabidopsis) or DISCORDIA1/ALTERNATIVE DISCORDIA1 (FASS/TON2 orthologs in monocots) are required for PPB formation. Knockout mutants lack PPBs and have incorrectly positioned division planes (Camilleri et al., 2002; Azimzadeh et al., 2008; Wright et al., 2009; Spinner et al., 2010). Thus, a potential mechanistic link between OFPs, OVATE-interacting TRMs and cell patterning is established through interactions with the TTP complex thereby regulating organ shape.

Whether the PPB is absolutely required for division plane patterning is not clear as cells in certain tissues appear to divide without TON1a and PPB formation in other tissues (Zhang et al., 2016a; Costa, 2017). Further insights about the function of the PPB and certain TRMs show that *TRM7* is a specific PPB marker whereas *TRM6* and *TRM8* are constitutively expressed throughout the cell cycle (Schaefer et al., 2017). The frequency of normal PPB formation is reduced in *trm7* mutants and no PPBs are found in the *trm678* triple mutants. Cells with disrupted PPB formation retain the capacity to define a cortical division zone but lose precision in the orientation of this division zone. Intriguingly, mutant *trm678* plants are fertile with normal organs that do not exhibit aberrant cell division patterns. The phragmoplast-orienting kinesin 1 (POK1) is a factor controlling the timing and efficiency of the cortical division zone (Lipka et al., 2014). In *trm678* mutant cells, even though POK1 targeting to the cell cortex is altered in the absence of the PPB, POK1 still forms a cortical ring that corresponds with the division zone (Schaefer et al., 2017). These results suggest that the PPB may be less of a causal determinant of the cell division plane and more of a regulator to ensure the fidelity of a division plane defined by another mechanism. Regardless, the position of the cell division plane has profound impact on the shape of plant organs, and therefore, much remains to be discovered of how plane positioning is regulated.

## SUN/IQD and the Cytoskeleton

The tomato SUN/AtIQD12 is a member of the IQ67 domain (IQD) protein family (Xiao et al., 2008; Huang et al., 2013). The IQ67 domain of IQD proteins is a conserved region of 67 amino acids and contains up to three regularly spaced IQ motifs which promote calmodulin (CaM) binding in the presence of Ca^2+^ (Rhoads and Friedberg, 1997; Abel et al., 2005, 2013). Ca^2+^ is a common secondary messenger in all eukaryotes and is used to regulate many cellular processes in response to both cellular and environmental stimuli, including cell division and shape (Cardenas, 2009; Steinhorst and Kudla, 2013; Burstenbinder et al., 2017b). The IQ67 domain of several IQD proteins interacts with CaM demonstrating that this family of proteins may serve as a large class of CaM binding proteins in plants (Burstenbinder et al., 2013, 2017b; Yang et al., 2018). The founding member of the IQD family, IQD1, localizes to microtubules with CaM2 in Arabidopsis and both IQD1 and IQD20 were found to interact with CaMs by Y2H, suggesting that IQDs may integrate Ca^2+^ sensing in regulation of the cytoskeleton (Levy et al., 2005; Burstenbinder et al., 2013).

Expression analyses in *N. benthamiana* showed that the N-terminus of most IQD proteins localizes to microtubules and that half of the IQDs localize to the plasma membrane (Burstenbinder et al., 2017b). There is also evidence that certain IQDs have differential subcellular localization dependent on the cell cycle stage (Wendrich et al., 2018). Cells in plants overexpressing *AtIQD16* had altered orientation of cMTs with more oblique aligned microtubules and significantly elongated cells. Colocalization of IQDs with CaM also suggests that IQD proteins are capable of sequestering or recruiting CaM to specific subcellular domains. Subcellular localization of several Arabidopsis IQDs (IQD12, IQD22, IQD24, and IQD25) showed punctate structures that are reminiscent of regions within the plasma membrane, which may act as signaling centers in the cell. *IQD14* is the rice ortholog to the Arabidopsis IQD15-18 subclade and loss of function alleles result in shorter and wider grains than wild type rice (Yang et al., 2018).

OsIQD14 was found to localize to the nucleus and cytoplasm and also in punctate locations along the microtubules, suggesting that the protein may function at specific points in microtubule regulation or on particular microtubule structures. Interestingly, expression of rice IQD14-GFP N- or C-terminal regions in *N. benthamiana* showed that the C-terminal region localized to the microtubules while the N-terminal region localized to the nucleus. This result is similar to the localization observed in the Arabidopsis IQD15-18 clade where the full length IQD protein was found on both microtubules and in the nucleus (Burstenbinder et al., 2017b; Yang et al., 2018). Rice IQD14 was also found to interact with Arabidopsis SPR2 by Y2H, and the orthologous Arabidopsis IQD15-18 subclade members were found to interact with SPR2 and CaM as well (Wendrich et al., 2018; Yang et al., 2018). SPR2 is a microtubule binding protein involved in protecting the minus end of microtubules and promoting severing and reorientation of the cMT arrays. SPR2 generally localizes to microtubules and does not distinguish dynamic from stable microtubules. However, IQD proteins may serve to direct the location of SPR2 function to specific regions to regulate reorganization the cytoskeleton in response to a certain signal (Buschmann et al., 2004; Shoji et al., 2004; Yao et al., 2008; Nakamura et al., 2018).

Auxin has been suggested to influence MT dynamics, but the mechanism is unclear. However, recent studies suggest that auxin-mediated cytoskeletal changes may involve IQD proteins (Chen et al., 2014; Wendrich et al., 2018) and IQDs are likely downstream targets of AUXIN RESPONSE FACTOR5/ MONOPTEROS (Boer et al., 2014; Moller et al., 2017). It has been proposed that IQDs in low auxin/ Ca^2+^ environments do not bind to CaM and instead bind to SPR2, inhibiting its function. This results in stabilized microtubules and a less dynamic cytoskeleton (Leong et al., 2018; Nakamura et al., 2018). Auxin leads to an increase in Ca^2+^, which promotes CaM binding to the IQD, and CaM binding then prevents IQDs from binding to SPR2. Unbound SPR2 can then bind the minus end of microtubules and promote microtubule branching and changes to cytoskeletal architecture in response to auxin (Wendrich et al., 2018). IQD proteins may function to resolve these signals and changes in Ca^2+^ levels within developing tissues in response to the environment, thereby directing cell elongation and expansion to ultimately drive organ shape.

As organ shape is controlled by both cell division and directed cell expansion, the regulation of microtubule dynamics is important in both of these processes to determine morphology. Some IQD proteins regulate cytoskeletal architecture by guiding the formation of ROP domains in the plasma membrane. ROPs are plant specific Rho GTPases with diverse functions (Yalovsky, 2015). One function of ROPs is in organizing the microtubule and actin cytoskeleton to determine a cell’s final shape. Some ROPs have been shown to promote aggregation of fine actin filaments and inhibit the assembly of organized microtubule arrays (ROP2 and ROP4), while ROP6 has been shown to have the opposite role and promotes accumulation of organized microtubule arrays (Fu et al., 2005, 2009; Ivakov and Persson, 2013). Furthermore, downstream of ROP6, the microtubule severing protein katanin is activated and promotes microtubule reorganization (Hamant, 2013; Lin et al., 2013). Formation of distinct subcellular domains of ROPs with these opposing functions can alter cytoskeleton composition and fine-tune the overall cell shape (Fu et al., 2002, 2005, 2009; Lin et al., 2013). The plasma membrane localization of Arabidopsis IQD13 has been shown to regulate ROP function in the xylem by promoting cMT growth and interaction with the membrane surface, thus restricting the formation of ROP11 domains (Oda and Fukuda, 2012; Sugiyama et al., 2017). In the presence of IQD13, the active ROP11 is restricted within narrow plasma membrane domains where it can recruit additional proteins to ultimately deplete the region of cMTs and form narrow pits in the secondary cell wall. In the absence of IQD13 or in the presence of truncated IQD13 lacking the plasma membrane associated domain, ROP11 forms circular domains that are independent of the cMTs and ultimately forms round pits in the secondary cell wall (Sugiyama et al., 2017). Secondary cell wall pit architecture is further refined by an interplay between the restriction of ROP11 to narrow domains by IQD13 and the impairment of ROP11 restriction and the resulting delineation of cMTs by CORTICAL MICROTUBULE DISORDERING1 protein (CORD) (Sasaki et al., 2017). AtIQD5 has recently been shown to regulate microtubule dynamics that affect cMT organization and subsequent cell shape formation in leaf pavement cells. IQD5 is enriched in lobed regions of pavement cells where cMTs are organized in parallel arrays and cell expansion is restricted (Liang et al., 2018; Mitra et al., 2018). In *iqd5* mutant cells, regions lacking IQD5 expression no longer form lobes and have altered cellulose deposition (Mitra et al., 2018). In summary, these results suggest that IQDs may regulate microtubule organization in distinct subcellular regions through interactions with ROPs in order to impact a cells final shape and ultimately organ shape.

Since organ shape is also influenced by anisotropic cell expansion (Maugarny-Cales and Laufs, 2018), the regulation of this expansion is an important factor controlling morphology. While cell expansion is driven by isotropic turgor pressure, the direction of expansion is controlled by the pattern of cellulose microfibrils, which are generally deposited perpendicular to the axis of expansion. This pattern resists turgor driven expansion in the direction parallel with the cellulose microfibrils, thus promoting expansion in the perpendicular direction (Szymanski and Cosgrove, 2009; McFarlane et al., 2014). cMTs regulate the deposition pattern of cellulose microfibrils within the cell wall by interacting with cellulose synthase complexes in the plasma membrane (Paredez et al., 2006; McFarlane et al., 2014). Therefore, the organization of microtubules, in part controlled by IQDs also impacts the arrangement of cellulose microfibrils which will impact how the cell wall expands and the ultimate shape of the cell. While randomly aligned cMTs are located in the cytoplasm away from the plasma membrane, cMTs closely anchored to the plasma membrane are organized in parallel bundles to preferentially serve as tracks for cellulose synthase movement (Barton et al., 2008). These cMTs rarely display lateral displacement from their parallel organization due to their tight association with the plasma membrane (Shaw et al., 2003). Cellulose microfibrils are synthesized by large CSCs, composed of 18–36 cellulose synthase subunits and their accessory proteins (McFarlane et al., 2014). Cellulose synthases are assembled and matured in the Golgi and sorted by the *Trans* Golgi network into small cellulose synthase compartments/ microtubule-associated cellulose synthase compartment vesicles for its secretion to the plasma membrane (Crowell et al., 2009; Gutierrez et al., 2009; Sampathkumar et al., 2013; Zhang et al., 2016b). CSCs are tethered to cMTs through cellulose synthase interactive protein 1 (CSI1) (Gu et al., 2010; Bringmann et al., 2012; Li et al., 2012; Mei et al., 2012), which determines their trajectory along the cMTs (Paredez et al., 2006). Delivery to the plasma membrane is also mediated by CSI1 and fusion with the plasma membrane is mediated by the plant specific protein PATROL1 and the exocyst complex (Zhu et al., 2018). AtIQD1 interacts with KLCR1 in Y2H and *in planta* where its recruitment to microtubules is dependent on IQD1 (Abel et al., 2013; Burstenbinder et al., 2013). KLCR and cellulose-microtubule uncoupling (CMU) are in the same protein family (Burstenbinder et al., 2017a) and the same proteins in Arabidopsis where At4g10840 encodes KLCR1 and CMU1 and At3g27960 encodes KLCR2 and CMU2 (Burstenbinder et al., 2013; Liu et al., 2016). CMU1 and CMU2 are localized as static puncta along microtubules. Disruption of CMU function causes lateral microtubule displacement. This compromises microtubule-based guidance of CSCs leading to cell twisting and altered growth (Liu et al., 2016). Together these results indicate that IQD proteins regulate how microtubules direct CSCs and the pattern of cellulose microfibril deposition, which influences cell expansion and organ shape. It is intriguing that AtOFP4 also regulates cell wall formation through its interaction with KNAT7 (Li et al., 2011). Thus, both IQDs and OFPs may influence organ shape at the cellular level through their regulation of cell wall formation.

With respect to the regulation of organ shape by SUN/IQDs and the role in cell division patterning, these proteins may directly influence this process. AtIQD5 is localized to the PPB, spindle, and phragmoplast of dividing root cells (Liang et al., 2018). ROPs may also be involved in PPB formation (Oda, 2018) because two ROP GTPase activating proteins interact with POK1 and are required for accurate orientation of the PPB, phragmoplast, and cell plate (Stockle et al., 2016). POK1 is required for division plane maintenance (Lipka et al., 2014; Stockle et al., 2016) and its function is also influenced by TRMs (Schaefer et al., 2017). It is plausible that SUN/IQD and TRM proteins coordinate cell division planes, which contribute to organ shape.

## Conclusion

A model to describe organ shape in the context of interactions of SUN/IQD, OFP and TRM, and associations with microtubules is shown in Figure 4. Assuming round shape as the default, the down regulation of OFP would lead to the association of more TRMs to the microtubules and hence elongated shape. This organ shape might also be attained via a similar mechanism when up-regulating TRM (Figure 4). Conversely, up-regulation of OFP and down regulation of TRM would result in rounder or even flat shapes due to less microtubular association of TRMs. SUN/IQD proteins are often found at the microtubules where their interaction with SPR2 and CaM might lead to altered cytoskeleton activities. Higher expression of SUN/IQD would lead to more association with the microtubules and hence elongated shape. Together with findings in Arabidopsis and crop plants, further information has shown that OFPs, TRMs and SUN/IQDs impact microtubular activities, offering mechanistic insights into how the different shapes of plant organs are realized.

## Author Contributions

ML and EvdK wrote and edited the review with significant contributions and further edits from SW, AS, and YW.

## Conflict of Interest Statement

The authors declare that the research was conducted in the absence of any commercial or financial relationships that could be construed as a potential conflict of interest.

## Footnotes

1 http://www.upov.int/upovlex/en/upov_convention.html

2 https://www.bioversityinternational.org/fileadmin/bioversity/publications/Web_ version/572/ch01.htm

## Abbreviations

CAP350: centrosome associated protein 350
cMT: cortical microtubules
CMU: cellulose microtubule uncoupling
CSC: cellulose synthase complex
FOP: fibroblast growth factor receptor 1 oncogene partner
IQD: isoleucine glutamine domain
KLCR: kinesin light chain related
MTOC: microtubule organizing center
NIL: nearly isogenic line
OFD1: orofaciodigital syndrome 1
OFP: ovate family protein
POK: phragmoplast-orienting kinesin
PPB: preprophase band
ROP: rho-GTPase of plants
SPR: spiral
TRM: tonneau1 recruiting motif
TTP: tonneau1-TRM-phosphatase 2C
γ-TuRC: tubulin ring complex
